# A novel approach for prediction of tacrolimus blood concentration in liver transplantation patients in the intensive care unit through support vector regression

**DOI:** 10.1186/cc6081

**Published:** 2007-07-26

**Authors:** Stijn Van Looy, Thierry Verplancke, Dominique Benoit, Eric Hoste, Georges Van Maele, Filip De Turck, Johan Decruyenaere

**Affiliations:** 1 Ghent University, Department of Information Technology (INTEC), Gaston Crommenlaan 8, Ghent, Belgium; 2 Ghent University Hospital, Intensive Care Department, De Pintelaan 185, Ghent, Belgium; 3 Ghent University, Department of Medical Statistics, De Pintelaan 185, Ghent, Belgium

## Abstract

**Introduction:**

Tacrolimus is an important immunosuppressive drug for organ transplantation patients. It has a narrow therapeutic range, toxic side effects, and a blood concentration with wide intra- and interindividual variability. Hence, it is of the utmost importance to monitor tacrolimus blood concentration, thereby ensuring clinical effect and avoiding toxic side effects. Prediction models for tacrolimus blood concentration can improve clinical care by optimizing monitoring of these concentrations, especially in the initial phase after transplantation during intensive care unit (ICU) stay. This is the first study in the ICU in which support vector machines, as a new data modeling technique, are investigated and tested in their prediction capabilities of tacrolimus blood concentration. Linear support vector regression (SVR) and nonlinear radial basis function (RBF) SVR are compared with multiple linear regression (MLR).

**Methods:**

Tacrolimus blood concentrations, together with 35 other relevant variables from 50 liver transplantation patients, were extracted from our ICU database. This resulted in a dataset of 457 blood samples, on average between 9 and 10 samples per patient, finally resulting in a database of more than 16,000 data values. Nonlinear RBF SVR, linear SVR, and MLR were performed after selection of clinically relevant input variables and model parameters. Differences between observed and predicted tacrolimus blood concentrations were calculated. Prediction accuracy of the three methods was compared after fivefold cross-validation (Friedman test and Wilcoxon signed rank analysis).

**Results:**

Linear SVR and nonlinear RBF SVR had mean absolute differences between observed and predicted tacrolimus blood concentrations of 2.31 ng/ml (standard deviation [SD] 2.47) and 2.38 ng/ml (SD 2.49), respectively. MLR had a mean absolute difference of 2.73 ng/ml (SD 3.79). The difference between linear SVR and MLR was statistically significant (*p *< 0.001). RBF SVR had the advantage of requiring only 2 input variables to perform this prediction in comparison to 15 and 16 variables needed by linear SVR and MLR, respectively. This is an indication of the superior prediction capability of nonlinear SVR.

**Conclusion:**

Prediction of tacrolimus blood concentration with linear and nonlinear SVR was excellent, and accuracy was superior in comparison with an MLR model.

## Introduction

### Purpose

Tacrolimus blood concentrations demonstrate a wide intra- and interindividual variability. Therefore, monitoring of these concentrations remains an issue of pivotal importance to safeguard therapeutic efficacy and to manage the risk for nephrotoxicity, other toxicities, and rejection in liver transplantation patients [1]. This study examines the feasibility and clinical benefits of using a support vector regression (SVR) algorithm in comparison with a multiple linear regression (MLR) algorithm in predicting tacrolimus blood concentration. Tacrolimus blood concentration is predicted starting from a selected number of clinically relevant input variables.

### Background

Hospital information systems in intensive care medicine generate large datasets on a daily basis. These rapidly increasing amounts of data make the task of extracting correct and relevant clinical information from intensive care unit (ICU) patients difficult [2,3]. Data modeling techniques based on machine learning such as support vector machines (SVMs) can partially reduce workload, aid clinical decision-making, and lower the frequency of human error [4]. Fundamental research in clinical data modeling forms the basis on which later validation can be performed in multicentered clinical trials. This is the first study to use SVM for data modeling in the ICU domain. SVMs have been applied, however, in molecular biology [5-7], bioinformatics [8], as well as in genetics [9] and proteomics [10,11]. In cancer research, kernel methods (or SVM) have been used to predict malignancy in brain tumors [12,13] and also in staging certain forms of breast and prostate cancer [14,15]. In cardiology, heart valve disease has been predicted with SVMs, and in fundamental cardiology research, nucleotide polymorphisms of candidate genes for ischemic heart disease have been modeled by kernel methods [16,17]. Clinical decision-making has been compared for prospective performance with logistic regression and SVM [18]. In contrast with the absence of data concerning SVM applications in the ICU, artificial neural networks (ANNs) – as a less recent statistical learning technique – have been studied thoroughly in the ICU environment: they have been used for prediction of ICU mortality and prognosis in septic shock [19,20], clinical decision-making [21], and prediction of plasma drug concentrations [22]. Also, the management of infectious diseases [23], real-time analysis of hemodynamics [24], and research in cardiology [25,26] and oncology [27,28] have benefited from recent evolutions in artificial intelligence (AI) and ANN.

### Underlying theory

**Figure 1:**
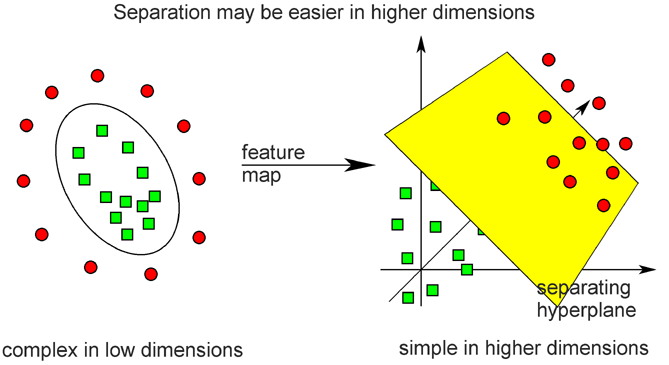
The support vector algorithm heuristic. In support vector machines, classification of datapoints or prediction of an outcome parameter is done by finding the 'hyperplane' that separates the datapoints by transforming the input variable dataset by a mathematical function into a 'higher dimension' in which separation is much easier (feature map = input variables dataset). The basis of this new heuristic is that classification of a seemingly chaotic input space is possible when one increases dimensionality and thereby finds a separating plane. Copyright permission from V.P. Bioinformatics (Improved Outcomes Software, Kingston, ON, Canada).

The roots of SVM lie in the statistical learning theory [29], which describes properties of learning machines which enable them to generalize well to unseen data. During the 1990s, SVM was developed by Vapnik and coworkers [30-32] at Bell Labs (formerly AT&T Bell Laboratories, Murray Hill, NJ, USA). A profound overview of the underlying theory and the SVM algorithm itself is given by Guyon and Elisseeff [33]. In the case of SVR [29], the goal is to find a function that predicts the target values of the training data with a deviation of at most ε, while requiring this function to be as flat as possible. The core of the support vector algorithm does this for linear functions *f*(*x*) = <*w*,*x*> + *b*, where (w,x) denotes the dot product of vectors w and x, thereby enforcing flatness by minimizing |w| (|w| denotes the Euclidian norm of vector w). By using a dual representation of the minimization problem, the algorithm requires only dot products of the input patterns. This allows the application of nonlinear regression by using a kernel function [34] that represents the dot product of the two transformed vectors. The MLR and the linear support vector algorithm are both linear approaches, but they differ in their underlying theoretical heuristics: the MLR method fits a model using the least-mean-squares heuristic (that is, the sum of the squared distances to the regression line is minimized). The support vector algorithm fits a flat-as-possible function by searching a separating hyperplane (Figure 1). The radial basis function (RBF) SVR method fits a nonlinear function onto the data, again aiming for maximum flatness. The RBF kernel is also often named a Gaussian kernel since the kernel function is the same as the Gaussian distribution function. Smola and Schölkopf [35] give an excellent overview of many details of the SVR procedure.

## Materials and methods

### Data

This study received approval from the Ethics Committee of Ghent University Hospital. Fifty patients who had recently undergone liver transplantation in Ghent University Hospital were included, and their medical records were reviewed. Tacrolimus blood concentrations, together with 35 other clinically relevant variables, were extracted from the ICU database. The following input variables were considered to influence tacrolimus blood concentration and were included: gender, age, weight, number of transplantations, number of days after surgery, existence of renal dysfunction (serum creatinine [CR] and urea [UR]) or liver dysfunction (alanine aminotransferase [ALT], aspartate aminotransferase [AST], gamma-glutamyl transpeptidase [GGT], total and conjugated bilirubin, alkaline phosphatise [ALKPHOS], and lactate dehydrogenase [LDH] levels), hematocrit (Hct), albumin, glucose, cholesterol, and six doses of tacrolimus, namely the dose at 8 a.m. and 8 p.m. from the three days (day 1, day 2, and day 3) before the day of the measured tacrolimus blood concentration (day 0). Coadministered medications were not included. Variables cholesterol and albumin were omitted due to too great a percentage of missing data (greater than 99%) (caused by not measuring these variables on a daily basis). The data were reorganized in patient days in which each record contained the following variables: gender, weight, age, days since transplantation, the 12 previously mentioned biochemical variables measured on day 0, the same parameters on day 1, tacrolimus blood concentration on day 1, the last six tacrolimus doses given, and the tacrolimus blood concentration on day 0 as a prediction target. This resulted in a total amount of 35 input variables and 1 output variable. Records in which the output parameter was missing were removed from the data. Patient days in which less than four of the six previous doses were available were also left out. This resulted in 457 records originating from 50 patients and a total of more than 16,000 data values. In these 457 records, 77% were complete, 15% contained a single missing value, and the remaining 7% had a maximum of 3 (of 35) values missing. This resulted in a total of 147/15,995 (0.92%) missing values. This extremely low number of missing values was filled in by means of an expectation maximization method [36].

### Data analysis

Data analysis for the linear SVR and the RBF SVR model was performed using software implemented by the authors based on the libSVM 2.82 [37] software package. Analysis for the MLR model was performed in SPSS 12.0 (SPSS Inc., Chicago, IL, USA). A mean absolute difference with the measured tacrolimus blood concentration of maximum 3 ng/ml and a standard deviation (SD) of maximum 5 ng/ml was agreed upon to be acceptable by expert opinion.

### Variable selection for the linear SVR and the RBF SVR model

This phase in the SVR model building is analogous with the variable selection phase for the MLR model. Using all 35 variables to construct a data model would result in suboptimal accuracy because different variables may contain overlapping information that disturbs the model-constructing process. Therefore, for each method (linear SVR, RBF SVR, and MLR), variable selection out of this total of 35 variables was done using recursive addition, recursive removal, stepwise addition, and stepwise removal of the input variables. These selection procedures are inspired by the commonly used stepwise regression technique in MLR, first presented by Effroymson [38]. The four selection procedures often result in different variable subsets. The best-performing subset was selected. In linear SVR, 15 features were selected: weight, age, days since transplantation, Hct, UR, ALKPHOS, ALT, total bilirubin, GGT (all on day 0), LDH on day 1, UR on day 1, morning doses of tacrolimus on day 2 and day 3, evening dose of tacrolimus on day 1, and the tacrolimus concentration on day 1. For RBF SVR, only two features sufficed: tacrolimus blood concentration on day 1 and the evening dose of tacrolimus on day 1. To validate a specific variable selection in linear SVR and RBF SVR, fivefold cross-validation was used. In this process, the available data are split into five equally sized parts. The remainder of the procedure is repeated five times. In each iteration, a different one of the five parts is kept apart, while the remaining four parts are used to construct the data model. The part that was kept separate is then used to verify the data model. The reported accuracy is the total of those measured in each of the five iterations, thus covering the total amount of available data.

### Variable selection for the MLR model

In the MLR model also, the variable selection was performed with a forward, a backward, and a stepwise algorithm for simple linear regression in SPSS 12.0 and regression coefficients were checked for significance. The significance level was set at α = 0.05. Adjusted *R*^2 ^values and goodness of fit were compared for the different MLR variable selections in SPSS. After selection of the final variable set for MLR, these variables were tested for correlation and multicollinearity. Variance inflation factor and eigenvalues were determined. For MLR, 16 variables were retained: gender, weight, age, Hct, LDH, UR, ALKPHOS, GGT, CR (all on day 0), AST on day 1, ALT on day 1, morning and evening doses of tacrolimus on day 1, evening dose of tacrolimus on day 2, and the morning dose of tacrolimus on day 3. Gender, weight, and age were included because of their clinical relevancy. After linear regression of this final variable set, normality testing of the residues as well as heteroscedasticity testing were performed. After searching the lambda value for the maximum likelihood with the Box-Cox algorithm, a transformation of the dependent variable (tacrolimus blood concentration) in the MLR model was performed because of heteroscedasticity of the residuals. To validate the final regression model, fivefold cross-validation was used, as in the SVR model.

### Parameter selection for the linear SVR and the RBF SVR model

Parameter selection denotes the process of setting data model parameters. These are the parameters that tune a data modeling technique. The MLR method has no such parameters. The linear SVR method has two such parameters: ε and C. Epsilon controls the flatness of the resulting data model, whereas C controls the cost of a prediction error: setting C to high values will result in fewer prediction errors in the training data. The RBF SVR method has three model parameters: the already discussed ε and C and the extra kernel function parameter γ, which determines the degree of nonlinearity: setting γ to high values results in a highly nonlinear data model [39]. The model parameters can be set using theoretical considerations that may assume certain properties of the data. The data, however, are not always perfect: it may contain noise and nonremoved trends. Parameter values obtained in this way are thus suboptimal. Therefore, in this study, the parameters are set using theoretical heuristics after which this initial setting is fine-tuned using pattern search [40]. To validate a specific parameter selection in linear SVR and RBF SVR, again fivefold cross-validation was used.

### Statistical analysis

Statistical analysis was carried out with SPSS 12.0. Results are reported as percentages, means, minimums and maximums, ranges, and SDs (as appropriate). A fivefold cross-validation algorithm was applied for validation of the prediction results. The correlation between measured and predicted tacrolimus blood concentrations was analyzed with a Spearman rank correlation coefficient. Differences between linear SVR, RBF SVR, and MLR were analyzed with the Friedman test and Wilcoxon signed rank test. A Bonferroni adjustment was performed for multiple testing. A Bland-Altman plot was used to illustrate significant differences between the three compared methods. Absolute difference as well as signed difference were studied. Mean absolute difference is the absolute difference between predicted and measured values, without its sign, and is an indication of the magnitude of the error, whereas mean signed difference indicates whether a model tends to predict higher or lower values than the measured value. The significance level was set at α = 0.05.

## Results

**Figure 2:**
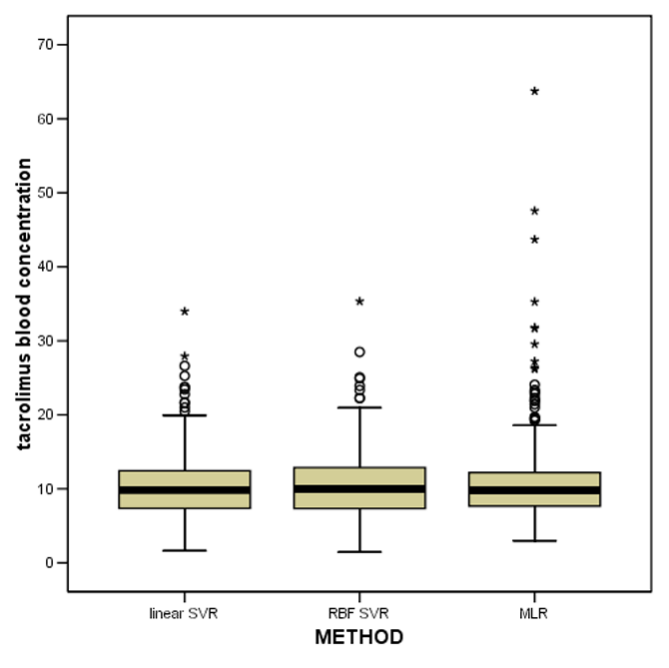
Outliers for the prediction of the tacrolimus blood concentration for the three models. MLR, multiple linear regression; RBF SVR, radial basis function support vector regression (nonlinear support vector regression); SVR, support vector regression. *'s represent extreme values (values more extreme than 3*IQR).

**Figure 3:**
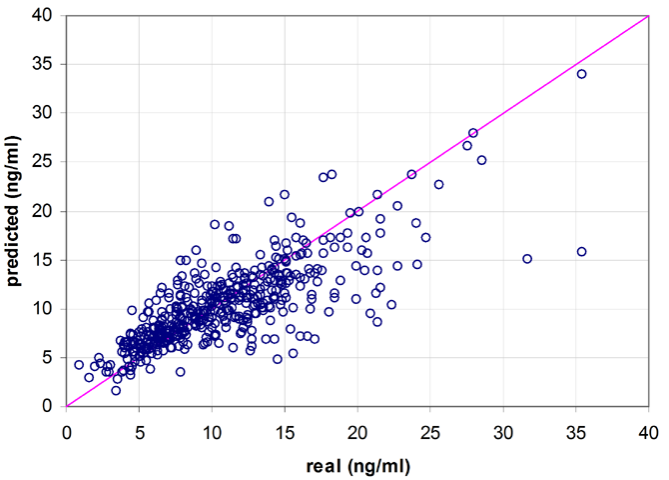
Correlation of real and predicted tacrolimus blood concentrations for the linear support vector regression model.

**Figure 4:**
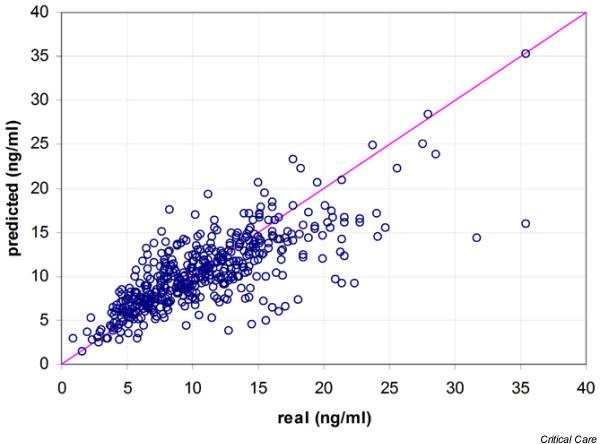
Correlation of real and predicted tacrolimus blood concentrations for the radial basis function support vector regression model.

**Figure 5:**
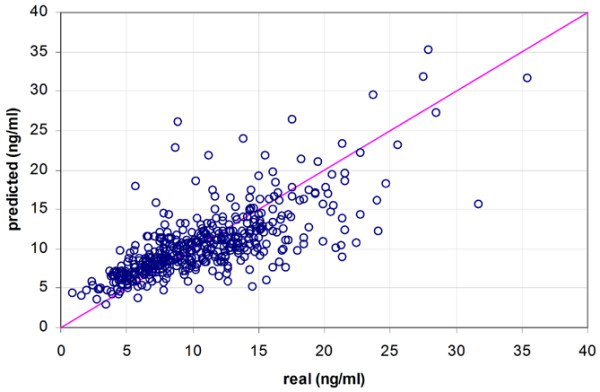
Correlation of real and predicted tacrolimus blood concentrations for the multiple linear regression model.

**Figure 6:**
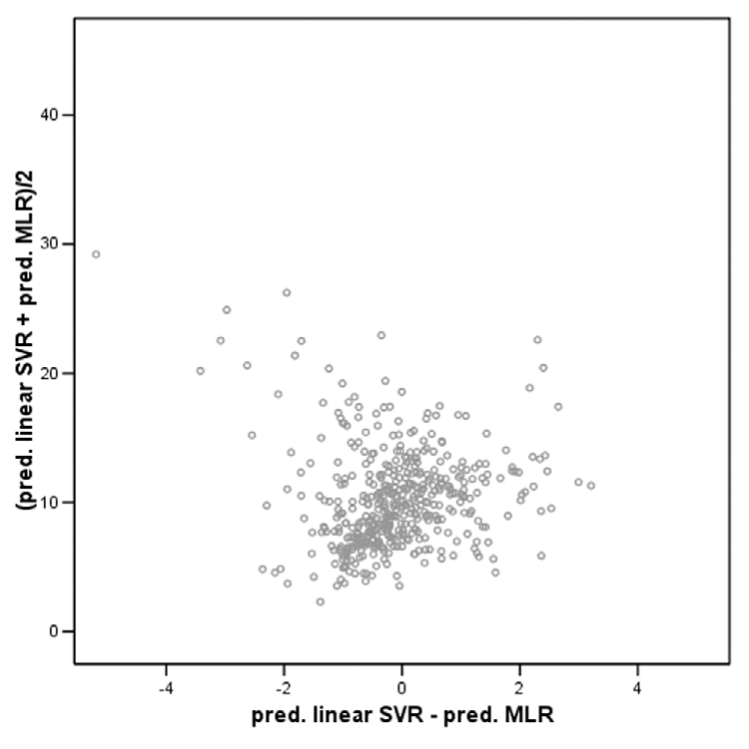
Bland-Altman plot for comparison of linear support vector regression (SVR) (a) and multiple linear regression (MLR) (b): x-axis = difference (linear SVR and MLR) = a - b; y-axis = mean of linear SVR and MLR = (a + b)/2. If two models are the same, all datapoints are represented around one point. However, in this figure, there is a considerable amount of spread between the datapoints of the linear SVR method and the MLR method which is a graphical illustration of the statistical difference found between these two methods.

Of the total study population, 58% (29/50) were male, mean age was 54 years (range 22 to 70), and mean weight was 79 kg. Table 1 gives a summary of the mean absolute differences between measured and predicted tacrolimus concentrations for the three models. In the distribution of the prediction errors made by the three methods, it has to be noted that the MLR model has the largest number of outliers (Figure 2). Figures 3 to 5 demonstrate the correlation between the observed tacrolimus blood concentration and the predicted blood concentration for linear SVR (Figure 3), RBF SVR (Figure 4), and MLR (Figure 5). These findings were corroborated by the Spearman rank correlation coefficients, which indicated good correlations for the three methods between the measured and the predicted blood concentrations: 0.762, 0.753, and 0.742 for linear SVR, RBF SVR, and MLR, respectively. Mean absolute difference between measured and predicted blood concentrations was smallest when using linear SVR: this difference between linear SVR and MLR was statistically significant (*p *< 0.001). Also, when mean signed differences were analyzed, the same significantly better results were observed in linear SVR in comparison with MLR. Even after *post hoc *analyses (α/3 for multiple testing, thus significance when *p *< 0.017), the significant difference between linear SVR and MLR remained valid. A Bland-Altman plot (Figure 6) outlines the difference between linear SVR and MLR.

**Table 1:** Predicted tacrolimus blood concentration and mean absolute difference between real and predicted tacrolimus blood concentrations

	Mean (ng/ml)	Standard deviation (ng/ml)	Minimum (ng/ml)	Maximum (ng/ml)
Predicted level linear SVR	10.4	4.2	1.6	34.0
Mean absolute difference	2.31	2.47	0.0	19.6
Predicted level RBF SVR	10.4	4.2	1.4	35.3
Mean absolute difference	2.38	2.49	0.0	19.4
Predicted level MLR	10.8	5.5	3.0	63.7
Mean absolute difference	2.73	3.79	0.0	54.4

MLR, multiple linear regression; RBF SVR, radial basis function support vector regression (nonlinear support vector regression); SVR, support vector regression.

## Discussion

Linear SVR for prediction of tacrolimus blood concentration resulted in a lower mean absolute error in comparison with the MLR model (Table 1). Incorporating nonlinearity in the predictor, however, by using a nonlinear kernel function, resulted in a prediction accuracy that was slightly less than in linear SVR, but this prediction still outweighed the accuracy of the MLR model. It is remarkable that this result was obtained using much fewer variables: only 2 input variables were used instead of 15 and 16 variables by the linear methods. Apparently, these 2 input variables contained more information in a nonlinear way than the other 15 or 16 contained in a linear way. When a linear method (linear SVR or MLR) was examined with only the 2 input variables used by the nonlinear RBF SVR model, the obtained prediction accuracy was lower than when using the nonlinear RBF SVR method. However, the added prediction strength of the extra 13 or 14 input variables in the linear SVR and MLR methods, respectively, is rather small. The nonlinear RBF SVR method is able to extract this extra information from only 2 input variables.

It is worth noting that in each of the three prediction models, the tacrolimus blood concentration on day 1 is incorporated, along with other variables. Obviously, the tacrolimus concentration on day 1 on its own already contains a lot of information about the level to be predicted. To verify the added value of incorporating this extra variable (tacrolimus dose on day 1), an RBF SVR model using only the previous tacrolimus blood concentration was constructed and evaluated using fivefold cross-validation. This model yielded a mean absolute error of 3.23 ng/ml (SD 3.12) and a maximum error of 26.33 ng/ml, indicating that adding the last evening dose of tacrolimus improves performance drastically. Moreover, it should be mentioned that in the linear kernel model a moderate amount of collinearity between the input variables was present (UR on 2 consecutive days, tacrolimus dose on 2 consecutive days) and that collinearity was not present between the two input variables of the RBF SVR model. In the MLR model, there was no problem of multicollinearity after the variable selection phase. It will be very interesting to see whether the results of this SVR model will be corroborated by similar results after testing this new technology on large multicentered ICU databases in future research.

This is the first report in which tacrolimus concentration is modeled by SVR, but a few other studies have already performed prediction of tacrolimus concentration using other AI techniques. Chen and colleagues [22] reported the use of a neural network and a genetic algorithm to predict the tacrolimus blood concentration. This neural network algorithm resulted in an average difference of the observed and predicted tacrolimus concentrations of 1.74 ng/ml with a range from 0.08 to 5.26 ng/ml. Bayesian forecasting as well has been applied in modeling tacrolimus concentrations. Fukudo and colleagues [41] demonstrated that Bayesian prediction of tacrolimus concentrations on the basis of previously acquired population-based pharmacokinetic data in adult patients receiving living-donor liver transplantation was possible within a certain timeframe after liver transplantation. However, a study by Willis and colleagues [42], using a population pharmacokinetic model based on Bayesian forecasting and adapted for individual pharmacokinetic, demographic, and covariate data, resulted in predictions that were too imprecise. In future research, the SVR-based model will be adapted to predict the tacrolimus dose to be given to ICU patients to obtain a predefined window of tacrolimus concentrations. Afterward, a randomized controlled trial will compare the accuracy of intensivists versus this SVR model in daily clinical practice.

## Conclusion

Results demonstrate a statistically significant superiority of linear SVR in comparison with MLR as well as a trend toward superiority of nonlinear SVR in comparison with MLR for the prediction of tacrolimus blood concentration in post-liver transplantation patients during ICU stay. The accuracies were all within clinically acceptable ranges. Moreover, nonlinear SVR required only two variables to make the tacrolimus blood concentration predictions. SVM technology has promising possibilities as a clinical decision agent in the ICU environment.

## Abbreviations

AI = artificial intelligence; ALKPHOS = alkaline phosphatise; ALT = alanine aminotransferase; ANN = artificial neural network; AST = aspartate aminotransferase; CR = serum creatinine; GGT = gamma-glutamyl transpeptidase; Hct = hematocrit; ICU = intensive care unit; LDH = lactate dehydrogenase; MLR = multiple linear regression; RBF = radial basis function; RBF SVR = radial basis function support vector regression (nonlinear support vector regression); SD = standard deviation; SVM = support vector machine; SVR = support vector regression; UR = urea.

## Competing interests

The authors declare that they have no competing interests.

## Authors' contributions

JD and FDT were responsible for the study concept, design, and overall responsibility. TV performed data acquisition and contributed to the statistical analysis and the drafting of the manuscript. SVL performed data transformation, wrote part of the SVM algorithm, and contributed to the drafting of the manuscript. DB and GVM contributed to the statistical analysis. All authors were responsible for the interpretation of data. All authors, including EH, contributed to the final manuscript. Funding for this study arose in part from project funding by an FWO scholarship and in part from clinical funding by the Ghent University Hospital. SVL and TV contributed equally to this article.
